# Short-term efficacy of intravitreal Aflibercept injections for retinal angiomatous proliferation

**DOI:** 10.1186/s12886-017-0497-0

**Published:** 2017-06-27

**Authors:** Hung-Da Chou, Wei-Chi Wu, Nan-Kai Wang, Lan-Hsin Chuang, Kuan-Jen Chen, Chi-Chun Lai

**Affiliations:** 1 Department of Ophthalmology, Chang Gung Memorial Hospital, Linkou, No. 5, Fuxing St., Guishan Dist., Taoyuan City, 333 Taiwan, Republic of China; 2 School of Medicine, Chang Gung University, No. 259, Wenhua 1st Rd., Guishan Dist., Taoyuan City, 333 Taiwan, Republic of China; 3 Department of Ophthalmology, Chang Gung Memorial Hospital, Keelung, No. 222, Maijin Rd., Anle Dist., Keelung City, 204 Taiwan, Republic of China

**Keywords:** Aflibercept, Age-related macular degeneration, Anti-angiogenic agents, Choroidal neovascularization, Retinal angiomatous proliferation, Type 3 neovascularization

## Abstract

**Background:**

To evaluate the short-term efficacy of intravitreal injections of aflibercept (IVA) to treat retinal angiomatous proliferation (RAP) and identify factors related to functional outcomes.

**Methods:**

This retrospective case series consisted of 19 eyes in 19 patients with RAP. All 19 eyes received 3 monthly consecutive IVA. The primary outcome measures were best-corrected visual acuity (BCVA) and central retinal thickness (CRT) after the last IVA.

**Results:**

Of the 19 treated eyes, 8 (42%) were pre-treated with 1 dose of bevacizumab one month prior to the initiation of treatment with aflibercept. BCVA was significantly improved and CRT was significantly reduced after 3 consecutive IVAs (*P* = 0.014 and *P* = 0.0002, respectively). Stabilization or improvement in BCVA was observed in 17 eyes (90%) treated with IVA. Eyes with baseline fibrovascular pigment epithelial detachment (PED) showed no significant gain in BCVA, and fibrovascular PED was negatively correlated with final BCVA (Spearman’s correlation coefficient = − 0.481, *P* = 0.037). The mean follow-up was 3.5 ± 0.5 months.

**Conclusions:**

In this short-term study, three consecutive IVAs showed efficacy for improving vision and reducing retinal edema in RAP patients. Eyes with fibrovascular PED showed poorer responses, and the presence of fibrovascular PED at baseline was negatively correlated with visual outcomes.

## Background

Retinal angiomatous proliferation (RAP) is recognized as a variant of neovascular age-related macular degeneration (nAMD) that displays a tendency to develop into a bilateral disease and has been associated with a guarded visual prognosis [1–3]. Cases of RAP comprise 15.1% of newly diagnosed cases of nAMD in Caucasians [4] and was identified in 4.5–7.5% of all Japanese nAMD patients [5, 6].

Before the introduction of anti-vascular endothelial growth factor (anti-VEGF) agents, treatments for RAP included conventional laser photocoagulation [7], surgical ablation [8, 9], transpupillary thermotherapy [10], photodynamic therapy (PDT) with verteporfin (Visudyne; Novartis Pharma AG, Basel, Switzerland) [11, 12], or combined intravitreal injections of triamcinolone acetonide with PDT [13]. These have each been studied and found to provide a limited response or have a high complication rate.

Intravitreal injections of anti-VEGF agents, such as bevacizumab (Avastin®, Genentech, South San Francisco, California, USA) and ranibizumab (Lucentis®; Genentech, South San Francisco, California, USA), have shown promising results for the treatment of RAP [14–16]. Intravitreal injections of aflibercept (IVA) (Eylea®; Bayer HealthCare, Berlin, Germany) have also been demonstrated to be effective for treating Type 1 and Type 2 nAMD [17]. However, to our knowledge, only a few studies have focused on responses to aflibercept in RAP patients [18–20]. The aim of this study was to determine the short-term efficacy of IVA as a treatment for RAP.

## Materials and methods

This was a retrospective case review of 19 eyes in 19 Taiwanese patients who presented with RAP between November 2013 and February 2016 in Chung-Gang Memorial Hospital, Taoyuan, Taiwan. The study was conducted according to the principles of the Declaration of Helsinki. The ethics committee of Chung-Gang Memorial Hospital approved this study. All patients were followed in a single center for at least 1 month after 3 monthly IVAs.

The diagnostic criteria for RAP lesions were based on clinical and angiographic findings [21], including 1) age of 55 years old or older; 2) nAMD with characteristics of intraretinal lesions when observed on optical coherence tomography (OCT), including associated intraretinal edema with or without sub-retinal pigment epithelium (RPE) fluid; 3) focal hyperfluourescent lesions and late leakage on fluorescein angiography (FA) at the site of an intraretinal lesion; and 4) a corresponding “hot spot” on indocyanine green angiography (ICGA). Other associated features more often observed in RAP [22, 23] were also analysed, including intraretinal hemorrhage (IRH) on fundus photography and near-infrared (IR) reflectance imaging, late leakage from the “hot spot” on ICGA late frames, identification of associated retinal feeding arterioles and draining venules on FA/ICGA, intraretinal cysts (IRC) and RPE interruption (PEI) along the PED on OCT, and the presence of reticular pseudodrusen (RPD) on OCT and IR imaging. All the clinical images were reviewed by a junior and a senior Ophthalmologist (HC and CL).

Patients with a defined RAP lesion who were treated with 3 consecutive monthly IVAs were included in the study. Patients with the following conditions were excluded: 1) a concurrent macular disease, such as diabetic maculopathy, retinal vascular occlusion, or macular telangiectasia; 2) any other condition that might affect a visual prognosis, such as uncontrolled glaucoma, degenerative myopia, trauma, uveitis, or previous vitreoretinal surgery; and 3) prior treatment with photodynamic therapy.

While the National Health Insurance Administration in Taiwan (NHI) covers the expense for 3 IVAs in nAMD patients, it takes a few weeks to obtain approval for these treatments. Therefore, to prevent further visual decline in patients, 8 eyes (42%, designated as the pre-treatment group) received 1 dose of intravitreal injection of bevacizumab (IVB) at the time of the RAP diagnosis and before IVA use was approved. This group of patients was required to pay for the cost of the IVB. One month later, after the NHI had approved the IVA application, each of these patients received three consecutive monthly IVAs. The remainder of the eyes (11 eyes, 58%, designated as the naïve group) were treatment-naïve and received three consecutive monthly IVAs after approval from the NHI.

The treatments were injected into the vitreous cavity 3.5 or 4.0 mm posterior to the corneal limbus using a 30-gauge needle after topical anaesthesia was applied, depending on the status of lens. The injection doses were 2 mg /0.05 ml for IVA and 1.25 mg/0.05 ml for IVB.

In every case, the baseline condition (at month 0) was examined before the first IVA treatment was performed. In the pre-treatment group, the baseline condition was examined 1 month after the IVB injection and before the first IVA treatment. The post-treatment condition of the eye was examined 1 month after the final injection of IVA (at month 3). All patients underwent a standardised examination including slit-lamp biomicroscopy, color and red-free fundus photography (TRC-50EX; Topcon, or Nonmyd α-DIII; KOWA, Tokyo, Japan), IR reflectance imaging and FA/ICGA (Heidelberg Retina Angiograph HRA2; Heidelberg Engineering, Dossenheim, Germany). OCT examinations were performed using spectral-domain OCT (SD-OCT) in a Spectralis HRA-OCT system (Heidelberg Engineering, Dossenheim, Germany). BCVA was measured using a standard decimal Landolt C visual acuity chart and converted to the logarithm of the minimum angle of resolution (LogMAR) equivalent for analysis. The CRT was defined as the distance from the RPE to the inner limiting membrane of the fovea center and measured using SD-OCT imaging and the software included with the machine.

The primary outcome measures were BCVA and CRT, which were determined using SD-OCT. The secondary outcome measures were the presence/absence of PED and subretinal fluid (SRF) on the SD-OCT after treatment was completed. The data are expressed as the mean ± standard deviation (SD).

Statistical analyses were performed using the Wilcoxon Signed-Rank Test to evaluate changes in BCVA and CRT. Bivariate relationships were examined using Pearson’s correlation analysis. The level of significance was defined as *P* < 0.05.

## Results

**Fig. 1:**
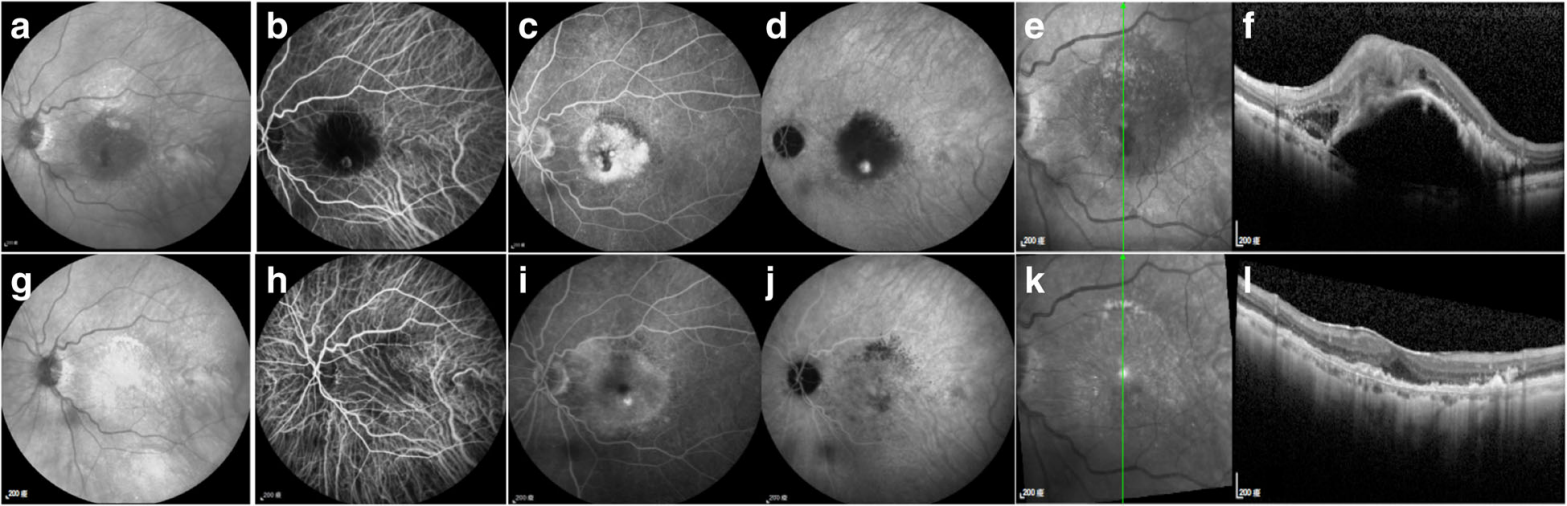
Intravitreal aflibercept treatment for RAP. Case 3: A 59-year-old female with a stage IIb RAP lesion. **a**-**f** Pre-treatment exams. **g**-**l** Post-treatment exams performed one month after 3 monthly IVAs. **a** Red-free fundus photograph showing a circumscribed area of PED with a focal area of retinal hemorrhage at the center. **b** Early phase ICGA demonstrating a neovascularised lesion in a macula with retino-retinal vessel anastomosis. **c** Late-phase FA showing dye leaking and pooling in the macula. **d** Late-phase ICGA revealing a “hot spot” corresponding to a neovascularised macular lesion. **e**, **k** The orientation of SD-OCT. **f** SD-OCT image taken before treatment showing a PED, subretinal fluid accumulation, and an overlying intraretinal lesion corresponding to the “hot spot” in late-phase ICGA. **g** Resolution of the circumscribed PED and the retinal hemorrhage. **h** The neovascularised lesion in the macula regressed. **i**,**j** Decreased dye leakage was observed in the macula, and the “hot spot” was no longer present. **l** Resolution of the PED and subretinal fluid. Some RPE bumps were still present

A total of 19 eyes in 19 patients (9 men and 10 women) were included (Table 1). All of the patients received 3 monthly IVA treatments. A subgroup of 8 patients (8 eyes, 42%) was pre-treated with 1 dose of IVB at 1 month before the initiation of the 3 monthly IVA treatments. The mean age of the patients was 68.2 ± 8.0 years old (mean ± SD, range, 57–82 years old). At baseline, 5 eyes (26.3%) had no PED, and 14 eyes (73.7%) had PED. 6 eyes (31.6%) had no SRF, and 13 eyes (68.4%) had SRF. All patients were followed up at least until 1 month after the last IVA treatment (Fig. 1).

**Table 1:**
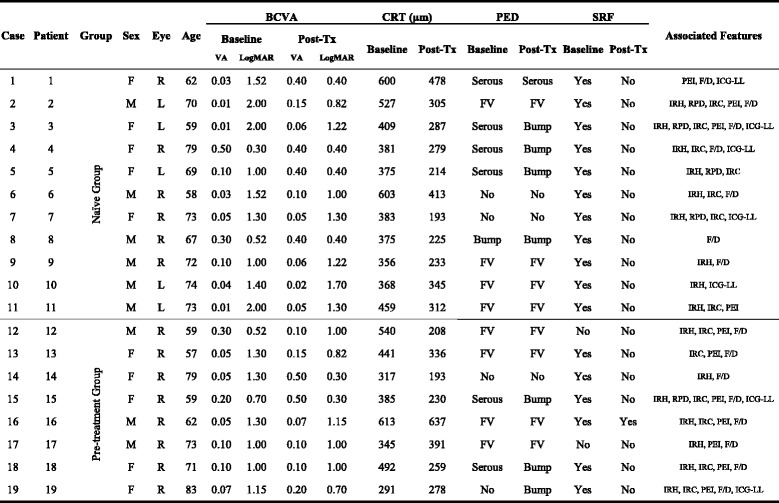
Baseline characteristics and clinical data before and after 3-monthly aflibercept injections

*BCVA* best-corrected visual acuity, *CRT* central retinal thickness, *F/D* feeding/draining vessels, *FV* fibrovascular, *ICG-LL* indocyanine green angiography late leakage, *IRC* intraretinal cyst, *IRH* intraretinal hemorrhage, *RPD* reticular pseudodrusen, *PEI* pigment epithelium interruption along the PED, *LogMAR* logarithm of the minimum angle of resolution, *PED* pigment epithelial detachment, *Post-Tx* post-treatment, *SRF* subretinal fluid, *VA* visual acuity

**Fig. 2:**
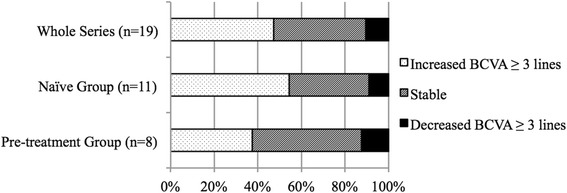
Graph showing the distribution of mean changes in best-corrected visual acuity (BCVA) from baseline after treatment with aflibercept. In the whole series, BCVA improved in 47.4%, remained stable in 42.1%, and decreased in 10.5% of the eyes. In the naïve subgroup, BCVA improved in 54.5%, remained stable in 36.4%, and decreased in 9.1% of the eyes. In the pre-treatment subgroup, BCVA improved in 37.5%, remained stable in 50.0%, and decreased in 12.5% of the eyes

Baseline BCVA was LogMAR 1.23 ± 0.56 (range, 0.30–2.00) and significantly improved to LogMAR 0.87 ± 0.41 (range 0.30–1.70) (*P =* 0.014) after treatment (Table 2). The mean change in BCVA was LogMAR 0.36 ± 0.56 (range, −0.48-1.70). The BCVA improved by 3 LogMAR lines or more in 9 eyes (47.4%), remained stable in 8 eyes (42.1%), and was worse by 3 lines or more in 2 eyes (10.5%) (Fig. 2).

**Table 2:** Functional and anatomical results in the whole series and in the 2 subgroups

		Baseline (mean ± SD)	Post-Tx (mean ± SD)	*P =* ^a^
Whole Series	BCVA (logMAR)	1.23 ± 0.56	0.87 ± 0.41	0.014
(*n* = 19)	CRT (μm)	404.0 ± 131.7	306.1 ± 112.0	0.0002
Subgroups				
Naïve Group	BCVA (logMAR)	1.32 ± 0.58	0.92 ± 0.47	0.047
(*n* = 11)	CRT (μm)	436.6 ± 96.6	298.6 ± 87.3	0.003
Pretreatment Group	BCVA (logMAR)	1.09 ± 0.54	0.78 ± 0.33	0.173
(*n* = 8)	CRT (μm)	359.1 ± 165.2	316.5 ± 145.4	0.018

*BCVA* best-corrected visual acuity, *CRT* central retinal thickness, *LogMAR* logarithm of the minimum angle of resolution, *Post-Tx* post-treatment, *SD* Standard deviation

^a^Wilcoxon Signed Ranks Test (2-tailed)

The baseline CRT mean ± SD was 404.0 ± 131.7 μm (range, 225–699 μm). CRT was significantly lower, at 306.1 ± 112.0 μm (range 193–637 μm) (*P =* 0.0002), after treatment (Table 2). The mean change in CRT was 97.9 ± 67.3 μm (range, 0–222.0 μm). When the mean change in CRT was compared to the mean baseline CRT (mean CRT change / mean baseline CRT), the mean percentage change in CRT was 23.1 ± 14.6% (range 0%–49.6%).

A subgroup analysis was performed to compare the naïve group and the pre-treatment group (Table 2). In the naïve group, the baseline mean ± SD BCVA was LogMAR 1.32 ± 0.58, and this value significantly improved to LogMAR 0.92 ± 0.47 (*P* = 0.047). In the same group, the baseline mean ± SD CRT was 436.6 ± 96.6 μm, and this value significantly decreased to 298.6 ± 87.3 μm (*P* = 0.003). In the pre-treatment group, the baseline mean ± SD BCVA was LogMAR 1.09 ± 0.54, and this value improved to LogMAR 0.78 ± 0.33, but the difference was not significant (*P* = 0.173). The baseline mean ± SD CRT was 359.1 ± 165.2 μm, and this value significantly decreased to 316.5 ± 145.4 μm (*P* = 0.018).

In the naïve group, the BCVA improved by 3 lines or more in 6 eyes (54.5%), remained stable in 4 eyes (36.4%), and decreased by 3 lines or more in 1 eye (9.1%) (Fig. 2). In the pre-treatment group, the BCVA improved by 3 lines or more in 3 eyes (37.5%), remained stable in 4 eyes (50.0%), and decreased by 3 lines or more in 1 eye (12.5%).

Overall, 14 eyes had baseline PED, 6 eyes had serous PED, and 8 eyes had fibrovascular PED. After treatment, PED had resolved in 5 eyes (35.7%). In the 6 eyes with baseline serous PED, PED resolved with only some RPE bump remained in 5 eyes (83.3%), and persistent PED was noted in 1 eye (13.7%). Fibrovascular PED persisted after IVA in all 8 of the eyes with baseline fibrovascular PED. No eyes without initial PED developed PED after treatment or during the follow-up period. A further subgroup analysis showed that patients with baseline fibrovascular PED had a poorer response to aflibercept and achieved no significant gain in BCVA (*P* = 0.446). The presence of fibrovascular PED at baseline was also negatively correlated with BCVA at the end of the follow-up period (Spearman’s correlation coefficient = − 0.481, *P* = 0.037).

SRF was present at baseline in 13 eyes (68.4%). After treatment with aflibercept, SRF had diminished completely in 12 (92.3%) of the 13 eyes with baseline SRF. No eyes without initial SRF developed SRF after treatment or during the follow-up period.

No ocular complications, including elevated intraocular pressure, ocular infection, drug-related intraocular inflammation, vitreous hemorrhage, or retinal detachment, were noted in any of the patients during the follow-up period. There were also no significant systemic side effects.

## Discussion

In this retrospective study, we show that IVA significantly achieved functional and anatomical improvements in RAP patients over a short-term follow-up period. Visual acuity improved in 47.4% of the eyes and stabilized in 42.1% of the eyes. CRT significantly decreased after IVA, SRF diminished in 92.3% of affected eyes, and PED resolved in 35.7% of affected eyes.

The best treatment option for RAP patients remains unclear. Few relevant studies are available, and their results are difficult to compare because of their small sample sizes and difference in their study designs. PDT monotherapy with verteporfin provides some improvements in visual response but has a high chance of causing RPE tears, especially in patients with larger RAP lesions [11, 12, 24]. Intravitreal injections of bevacizumab or ranibizumab have been investigated and found to be effective for improving or maintaining BCVA [14, 25–27]. A head-to-head comparison between intravitreal injections of ranibizumab (IVR) and IVB was conducted, and the results showed that these two anti-VEGF agents were equally effective in improving or maintaining visual acuity in RAP patients [28]. In addition, after IVR, the rate of developing RPE tears was lower than those treated with PDT monotherapy [29], but the rate of geographic atrophy was higher than in patients with typical nAMD [30].

Aflibercept had been demonstrated to be as effective as ranibizumab in treating nAMD, and required fewer injections [17]. IVA was also associated with a higher incidence of drying the macula than IVR in patients with choroidal vascular hyperpermeability [31], and associated with a higher reduction rate of PED in nAMD patients [19]. In nAMD eyes that showed a poor response to IVR, switching to IVA achieved functional and anatomical improvements [32, 33].

However, study focused on the effect of treating RAP patients with aflibercept is scarce, with small case numbers and limited information on outcomes [19, 20]. Recently, Matsumoto et al. [18] reported the one-year results of treat-and-extend regimen of IVA on treating RAP. In their series, BCVA significantly improved and CRT significant decreased since post-IVA month one and persisted up to one year. However, the responses of PED were not described.

In the current study, 73.7% of the eyes had PED at baseline, and the mean baseline BCVA was relatively poor (Snellen chart equivalent of 20/340). Both the anatomical and functional responses to IVA were favorable, and the post-treatment mean BCVA significantly improved to 20/148, while the mean CRT was reduced from 404.0 μm to 306.1 μm. These results imply that aflibercept effectively dried the retina and improved vision even in patients with advanced RAP lesions and poor visual acuity. Nevertheless, the current study outcomes represent only short-term results, and RAP lesions are notorious for their high recurrence rate. Hence, a study including a longer observation period is needed to more accurately determine the long-term effects of IVA.

The reason that BCVA did not significantly improve in the pre-treatment subgroup is unclear. The fact that the rate of fibrovascular PED was initially higher (50%) in the pre-treatment group than in the in the naïve group (36%) could have contributed to the poorer responses that were observed in the pre-treatment group. The fact that baseline BCVA was better in the pre-treatment group may also have left less room for improvement: BCVA tended to be maintained (50.0%) in the pre-treatment group and improved (54.5%) in the naïve group. In both groups, only 1 eye had a BCVA that worsened by more than 3 lines.

PED, and especially fibrovascular PED, was a more difficult condition to manage. In previous reports on nAMD, PED has been associated with a poorer prognosis in patients treated with anti-VEGF agents [34, 35]. One recent report concluded that in patients with nAMD, fibrovascular PED had a lower probability of resolving than serous PED [36]. In the current series, the only 2 cases in which BCVA worsened by >3 lines (case 10 and 12) had baseline fibrovascular PED that persisted after IVA, and this prevented them from showing improved BCVA. The only 2 cases that had a thicker CRT after IVA (case 16 and 17) also had baseline fibrovascular PED, but their BCVA remained stable after treatment. Tsaousis et al. [37] reported 2 eyes in which CRT decreased while BCVA failed to improve. In our series, 4 eyes displayed reduced CRT but worsened BCVA. All 4 of these eyes had initial PED.

The above findings suggest that patients with PED lesions are at risk of having PED progression and altered RPE function. Both of these effects could impede visual outcomes despite the retina-drying effect of aflibercept. Although aflibercept has been reported to have better efficacy in resolving PED in nAMD patients than ranibizumab [36, 38, 39], the appropriate treatment for patients with RAP that has advanced beyond stage IIb remains to be determined in future studies.

Limitations of this study include its retrospective design and short-term follow-up period. Nearly half of the included patients were pre-treated with IVB, and this may have altered the conditions in the pre-treatment group. Although the period of clinical activity of bevacizumab has been reported to be as long as 100 days [40], and although a 6 weekly evaluation/treatment interval was proposed [41], most current study protocols use a series 3 monthly loading injections of bevacizumab to achieve an initial clinical response [42]. In our study, IVB dried the serous PED in 1 eye and improved mean CRT from 428 ± 165.2 μm (before IVB, referred to as month −1) to 359.1 ± 165.2 μm (at 1 month after IVB, referred to as month 0). However, this change was not significant (*P* = 0.263). It was only after IVA that the mean CRT in the pre-treatment group significantly improved. These improvements might be partially related to the effect of bevacizumab, but aflibercept appears to play a major role. The small sample size of the cohort may also have compromised data analysis. A study including a control group or that evaluates a matched comparison between different anti-VEGF agents is needed to clarify this issue. A longer follow-up period is also critically needed to evaluate the incidence of late complications, including geographic atrophy and RPE tears, and to determine the closure rate of vessel anastomosis.

## Conclusion

Our study shows that IVA was efficacious in treating RAP patients over a short-term follow-up period. Ninety percent of the eyes showed improved or stable BCVA at the end of follow-up. Patients with baseline PED, especially fibrovascular PED, were more difficult to manage. Further studies are needed to determine the long-term efficacy of IVA in RAP patients in different stages of the disease.

## Abbreviations

anti-VEGF: anti-vascular endothelial growth factor
BCVA: best-corrected visual acuity
CRT: central retinal thickness
F/D: feeding/draining vessels
FA: fluorescein angiography
ICGA: indocyanine green angiography
IR: near-infrared
IRC: intraretinal cysts
IRH: intraretinal hemorrhage
IVA: intravitreal injections of aflibercept
IVB: intravitreal injection of bevacizumab
IVR: intravitreal injections of ranibizumab
LogMAR: logarithm of minimum angle of resolution
nAMD: neovascular age-related macular degeneration
NHI: National Health Insurance Administration in Taiwan
OCT: optical coherence tomography
PDT: photodynamic therapy
PED: pigment epithelial detachment
PEI: RPE interruption
RAP: retinal angiomatous proliferation
RPD: reticular pseudodrusen
RPE: retinal pigment epithelium
SD: standard deviation
SD-OCT: spectral-domain OCT
SRF: subretinal fluid
