# Erratum to: short-term efficacy of intravitreal Aflibercept injections for retinal angiomatous proliferation

**DOI:** 10.1186/s12886-017-0531-2

**Published:** 2017-08-15

**Authors:** Hung-Da Chou, Wei-Chi Wu, Nan-Kai Wang, Lan-Hsin Chuang, Kuan-Jen Chen, Chi-Chun Lai

**Affiliations:** 1 Department of Ophthalmology, Chang Gung Memorial Hospital, Linkou, No. 5, Fuxing St., Guishan Dist, Taoyuan City, 333 Taiwan, Republic of China; 2 School of Medicine, Chang Gung University, No. 259, Wenhua 1st Rd., Guishan Dist, Taoyuan City, 333 Taiwan, Republic of China; 3 Department of Ophthalmology, Chang Gung Memorial Hospital, Keelung, No. 222, Maijin Rd., Anle Dist, Keelung City, 204 Taiwan, Republic of China

## Erratum

After publication of the article [1], it has been brought to our attention that Table 1 has been formatted incorrectly in the PDF version. The table should be clearly divided into two groups of patients. Patients 1–11 are in the “Naïve Group” and patients 12–19 are in the “Pre-treatment Group”. This has been updated to reflect this in the original version of the article.
